# Network Pharmacology and Molecular Docking Elucidate the Mechanism of Phillyrin in Colorectal Cancer

**DOI:** 10.1002/fsn3.71069

**Published:** 2025-10-23

**Authors:** Zu‐Jian Hu, Guang‐Jia Lv, Wei‐Song Dong, Fan Zheng, Peng‐Hua Yan, Shen‐Lei Yu, Dan‐Ni He, Bo‐Yang Liu, Yuan‐Yuan Gao, Dong‐Yan Su, Yang‐Yang Guo, Yong‐Heng Bai, Heng‐Yue Zhu

**Affiliations:** ^1^ Zhejiang Key Laboratory of Intelligent Cancer Biomarker Discovery and Translation The First Affiliated Hospital of Wenzhou Medical University Wenzhou Zhejiang China; ^2^ College of Life Sciences Northeast Forestry University Harbin Heilongjiang China; ^3^ Department of Pathology The First Affiliated Hospital of Wenzhou Medical University Wenzhou Zhejiang China

**Keywords:** colorectal cancer, molecular docking, molecular dynamics simulation, network pharmacology, phillyrin

## Abstract

Phillyrin is an important active component of the traditional Chinese medicinal herb *Forsythia suspensa* Vahl (Oleaceae) and has demonstrated anti‐inflammatory, antioxidant, and antitumor properties. However, there is a paucity of studies investigating its therapeutic efficacy in colorectal cancer (CRC). Our research aims to explore the potential therapeutic effects and underlying mechanisms of phillyrin in treating CRC. Phillyrin potential targets were predicted using the ChEMBL, HERB, and SwissTargetPrediction databases. TCGA and GEO databases were employed to acquire targets associated with CRC. Afterward, we used the STRING web database to perform network analysis of protein–protein interactions (PPI) on the shared genes. Key genes were discovered in the PPI network and subsequently analyzed using the Gene Ontology (GO) and Kyoto Encyclopedia of Genes and Genomes (KEGG) databases. Apoptosis analysis in colorectal cancer cells treated with varying amounts of phillyrin was conducted using flow cytometry. Additionally, western blot analysis was applied to assess the expression of PI3K/AKT signaling proteins, confirming the findings of the network pharmacology study. A total of 250 phillyrin target genes and 4414 CRC‐related targets were screened. Eight central genes were obtained through PPI network topological analysis. Analysis of GO and KEGG enrichment indicated that phillyrin is strongly linked to various signaling pathways, particularly the PI3K/AKT pathway. In vitro experiments, treatment with phillyrin at a concentration of 0.2 mM induced apoptosis rates of approximately 17% in HT29 cells and 21.1% in HCT116 cells. Cell migration was also significantly inhibited, with HT29 migration reduced to about 15.7% and HCT116 migration reduced to about 33.4% of the control after 24 h of treatment. Additional examination indicated that the PI3K/AKT/mTOR pathway plays a vital role in determining the effectiveness and outlook of phillyrin treatment in colorectal cancer. This study reveals that phillyrin inhibits CRC cell metastasis and induces apoptosis via the PI3K/AKT/mTOR pathway, highlighting its promise as a potential therapeutic candidate for future colorectal cancer treatment.

## Graphical abstract

Phillyrin, a natural compound from *Forsythia suspensa* , was investigated for its therapeutic potential in colorectal cancer (CRC). Network pharmacology identified key targets and pathways, with enrichment analyses highlighting the PI3K/AKT/mTOR axis. In vitro assays demonstrated that phillyrin suppressed CRC cell migration and induced apoptosis, supporting its potential as a novel candidate for CRC treatment.

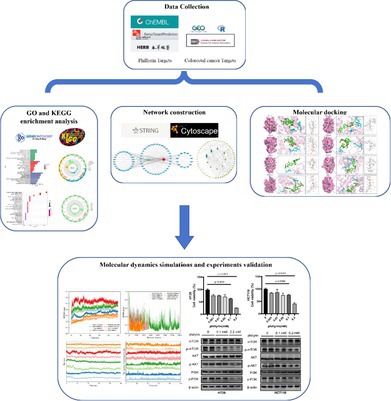

**Abbreviations**
BC: betweenness centrality
BP: biological processes
CC: cellular components
CC: closeness centrality
CCK‐8: cell counting kit‐8
CRC: colorectal cancer
CTD: compound‐Target‐Disease
DC: degree centrality
DEGs: differentially expressed genes
EC: eigenvector centrality
EMT: epithelial‐mesenchymal transition
GAFF: generalized AMBER force field
GEO: Gene Expression Omnibus
LAC: local average connectivity‐based method
MF: molecular functions
PPI: protein–protein interaction
Rg: radius of gyration
RMSD: root mean square deviation
RMSF: Root Mean Square Fluctuation
TCGA: the Cancer Genome Atlas

## 1 Introduction

Colorectal cancer is a common malignancy that impacts the gastrointestinal tract. According to the GLOBOCAN 2020 statistics, colorectal cancer (CRC) is the third most common cancer globally and the second leading cause of cancer‐related deaths. In China, CRC emerges as one of the top five prevalent malignancies, with an estimated 408,000 newly reported cases and approximately 195,000 associated fatalities (Sung et al. 2021). Although substantial progress has been made in systemic treatments such as biological therapy and cytotoxic chemotherapy for metastatic CRC, the situation for CRC patients remains grim (Zhong et al. 2023).

Herbs play a crucial role in traditional Chinese medicine. However, the complexity of their components can lead to some side effects, including organ failure, despite their significant therapeutic effects on diseases (Wu et al. 2019). Numerous bioactive ingredients have been isolated from herbs and have demonstrated efficacy in inhibiting tumor growth in both experimental and clinical studies (Karağaç et al. 2024; Kizir et al. 2024). *Forsythia suspensa Vahl (Oleaceae)*, a traditional Chinese medicine, possesses various functions such as sterilization, heat clearing, and detoxification (Jia et al. 2015). Being a complex natural product, *Forsythia suspensa* has also been found to actively participate in the biological regulation of tumor growth (Wang et al. 2018). Research has shown that, as a major bioactive component of *Forsythia suspensa* , phillyrin can suppress the development of laryngeal squamous cell carcinoma by blocking the AMPK/mTOR pathway (Wang et al. 2019). Furthermore, it has been suggested that dysregulation of the mTOR‐PI3K‐Akt pathway is closely associated with the risk of CRC (Simons et al. 2022; Zhou et al. 2021). Consequently, phillyrin represents a promising novel candidate for anticancer therapeutics.

The field of network pharmacology has emerged from the theory that compounds exhibiting selective activity toward multiple targets are likely to be more efficacious compared to drugs that target a single entity (Isıyel et al. 2025; Liu et al. 2021; Özturk et al. 2024). Investigating potential interactions between drugs and targets is increasingly utilized in drug discovery (Tokalı, Demir, Ateşoğlu, et al. 2025a; Tokalı, Demir, Çakır, et al. 2025). This approach aims to explore pharmacological mechanisms, adverse reactions, and resistance, with the goal of devising innovative therapeutic approaches (Shang et al. 2023). In the pursuit of more effective and safer drugs, it is essential to leverage ligands with established structures sourced from chemical literature or drug databases. Subsequently, elucidating the characteristics of their respective targets becomes crucial. In addition, network pharmacology offers an additional advantage in its heightened efficacy compared to conventional drug discovery methods in pharmaceutical development (Tokalı, Demir, Ateşoğlu, et al. 2025b; Tokalı, Demir, Şenol, et al. 2025).

This study investigates the potential therapeutic efficacy of phillyrin in CRC by employing network pharmacology to identify unexplored target pathways. This study aims to explore the effects and mechanisms of phillyrin on colorectal cancer. Network pharmacology is utilized to identify pertinent targets linked to CRC, followed by validation through the selection of targets demonstrating strong correlation and significant impact. This validation process encompasses both computational analysis and experimental verification. Overall, our research substantiates the anticancer properties of phillyrin in CRC.

## 2 Materials and Methods

### 2.1 Drugs and Reagents

Phillyrin (≥ 98.0%, HY‐N0482) was purchased from MedChemExpress. The antibodies used in the western blot assays were as follows: β‐actin (Affinity, AF7018, dilution ratio: 1:1000); AKT (Abcam, ab8805, dilution ratio: 1:1000); p‐PI3K (Affinity, AF3242, dilution ratio: 1:1000); p‐AKT (CST, 4060 s, dilution ratio: 1:1000); PI3K (Affinity, AF6241, dilution ratio: 1:1000); mTOR (Proteintech, 66888‐1‐Ig, dilution ratio: 1:1000); p‐mTOR (Affinity, AF3308, dilution ratio: 1:1000); Goat Anti Rabbit IgG‐HRP (Affinity, S0001, dilution ratio: 1:5000).

### 2.2 Screen of Phillyrin Targets

**FIGURE 1:**
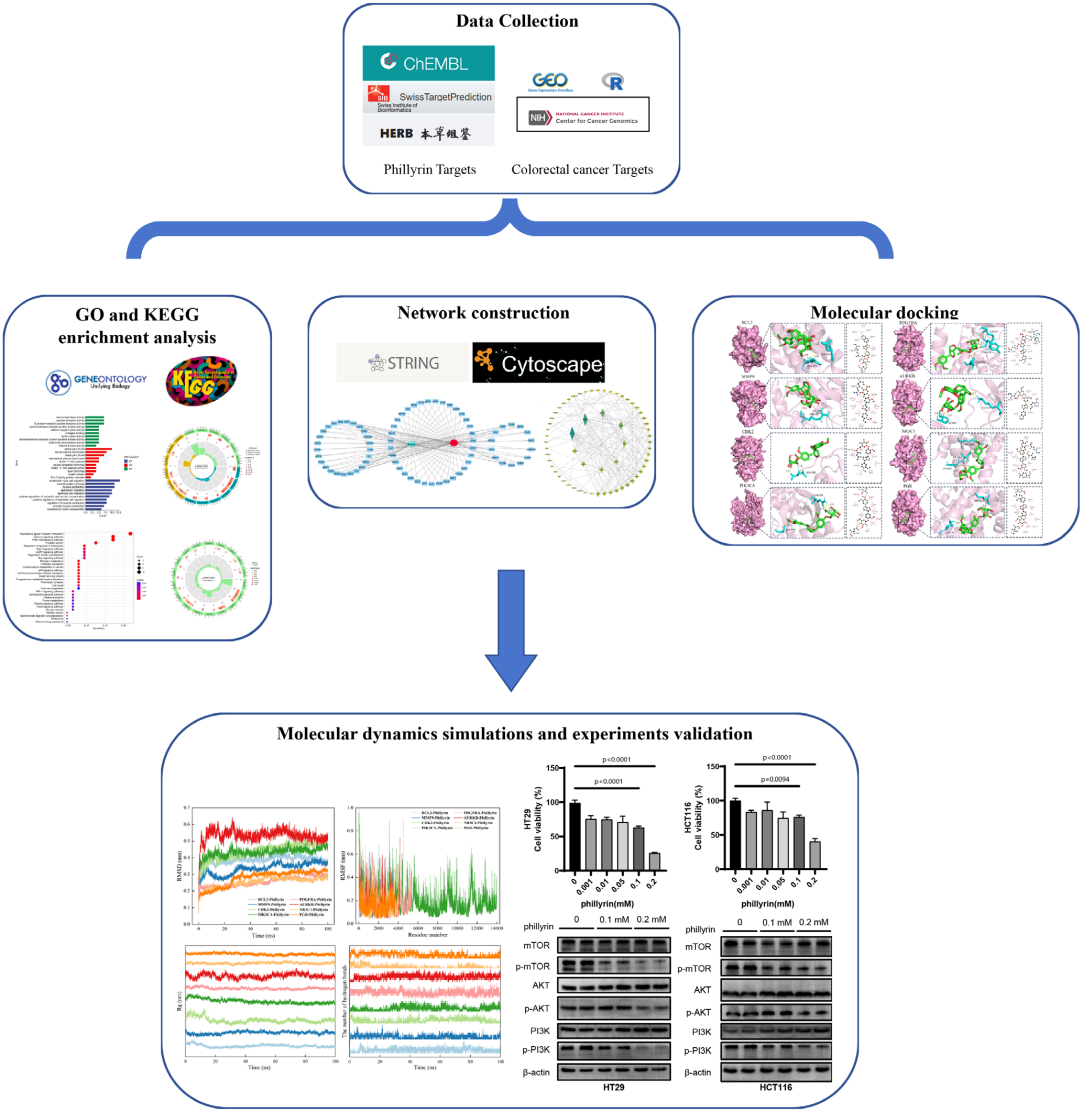
The flowchart to study the molecular mechanism of phillyrin for the treatment of colorectal cancer.

As presented in the workflow (Figure 1). Data on phillyrin's chemical properties were obtained from the PubChem database. Phillyrin candidates were gathered from three sources: the SwissTargetPrediction web server, the ChEMBL database, and the HERB database.

### 2.3 Identification of CRC Targets

Data on colorectal cancer (CRC) datasets were obtained from two different sources: the Cancer Genome Atlas (TCGA) and the Gene Expression Omnibus (GEO) database, specifically accession number GSE18105. Identifying genes associated with CRC involved identifying differentially expressed genes (DEGs) in various subgroups with a significance level of *p* < 0.05 and |logFC| > 2. Then, the DEGs in the TCGA and GSE18105 datasets were regarded as the targets for CRC.

### 2.4 Establishment of the Drug‐Target‐Disease Network

The intersecting target genes of phillyrin against CRC were obtained using the “VennDiagram” R package. The regulatory drug‐target‐disease network was mapped by the software Cytoscape in order to explain the relationship scientifically and rationally between compounds and targets.

### 2.5 Building a PPI Network and Discovering Key Candidate Targets

Phillyrin and CRC intersection targets were uploaded to the STRING 12.0 database at https://string‐db.org/ in order to create a PPI network. The confidence parameter was set to “medium confidence > 0.4,” and the organism was specified as “ *Homo sapiens* ”. Afterwards, the protein–protein interaction network was exported and displayed with Cytoscape version 3.7.0. The software utilized the cytoHubba plugin to identify central targets using five popular algorithms: degree centrality (DC), betweenness centrality (BC), closeness centrality (CC), eigenvector centrality (EC), and local average connectivity‐based method (LAC). This plugin is a powerful tool for core target screening, extracting the top 20 ranked targets from each algorithm and mapping them onto a Venn diagram. The intersection of these targets was identified as core candidate targets, enhancing the accuracy of the screening process.

### 2.6 GO and KEGG Signaling Pathway Enrichment Analysis

R (v. 3.4.2) was used to conduct and visualize GO and KEGG pathway enrichment analysis, using the packages “org.Hs.eg.db”, “clusterProfiler”, “AnnotationHub”, and “ggplot2”.

### 2.7 Molecular Docking and Molecular Dynamics Simulation

Molecular docking was performed using AutoDockTools software (v. 1.5.6) to investigate the interactions between Phillyrin and BCL2, MMP9, CDK2, PIK3CA, PDGFRA, AURKB, NR3C1, and PGR. The three‐dimensional structures of the proteins were obtained from the PDB database (https://www.rcsb.org/) and manually repaired for missing residues. The structure of Phillyrin was obtained from the ZINC database (http://zinc20.docking.org/) and hydrogenated using Avogadro software. Structural optimization was conducted using ORCA software with the B3LYP hybrid functional and 6‐311G** basis set. Blind docking was employed, and a docking box of 120 × 120 × 120 was established. The Lamarckian genetic algorithm was used for 50 docking runs (Kalay et al. 2025). Redocking of the control group was performed to confirm the docking accuracy (RMSD < 2 Å). Finally, the most stable conformations were analyzed using PyMOL and LigPlot.

Molecular dynamics simulations were performed using GROMACS 2020 with the AMBER99SB force field (Kalay et al. 2025; Kuzu and Demir 2025). Phillyrin was parameterized using the GAFF, and its RESP charges were calculated using ORCA and Multiwfn; the resulting charge data were incorporated into the topology file. The TIP3P water model was used for solvation, and periodic boundary conditions were applied to the simulation box. The equations of motion were integrated using the leap‐frog algorithm with a time step of 2 fs. The LINCS algorithm was used to constrain hydrogen bond lengths, with parameters lincs_iter = 1 and lincs_order = 4. Simulations were conducted under conditions of 300 K temperature and 1 bar pressure, with a time step of 2 fs and a total simulation duration of 100 ns. After the simulation, built‐in tools were used to analyze the trajectories and calculate the RMSD, RMSF, radius of gyration (Rg), and number of hydrogen bonds for each system.

### 2.8 Cell Culture

Human CRC cell lines HT29 and HCT116 were purchased from the Cell Bank of the Chinese Academy of Sciences (Shanghai, China). Cells were cultured in DMEM (Invitrogen, Carlsbad, CA, USA) with 10% fetal bovine serum and 1% penicillin–streptomycin. Cell lines were cultured in a humidified atmosphere with 5% CO_2_ at 37°C.

### 2.9 Cell Counting Kit‐8 (CCK‐8) Assay

Cell viability was assessed using the Cell Counting Kit 8 (CCK‐8) reagent obtained from GlpBio in Shanghai, China, according to the provided guideline (Su et al. 2022). HT29 and HCT116 cells in the phase of rapid growth were placed in a 96‐well dish. Then different concentrations of phillyrin (0, 0.1 mM, or 0.2 mM) were applied to treat cells for 24 h. Subsequently, an additional 10 μL of CCK‐8 reagent was introduced into each well, followed by an incubation period at 37°C for 2 h. The microplate reader was used to measure the absorbance at 450 nm, also known as OD 450 nm. All experiments were repeated independently at least three times.

### 2.10 Colony Formation Assay

The colony formation assay was conducted as previously reported to investigate cell proliferation (Guo et al. 2023). Cells were initially placed in a 6‐well plate at a concentration of 1000–1500 cells per well and left to incubate until colonies were visible. Subsequently, HT29 and HCT116 cells were treated with phillyrin (0, 0.1 mM, and 0.2 mM) for 24 h. Following this, the liquid in every well was substituted with standard complete DMEM, and the cells were grown for an extra 7–10 days prior to fixing and coloring cell clusters using 4% paraformaldehyde and 0.5% crystal violet. All experiments were repeated independently at least three times.

### 2.11 Apoptosis Assay

First, HT29 and HCT116 cells were seeded in the 24‐well plates at 5 × 10^4^ cells/well. Subsequently, apoptosis levels in cells exposed to different concentrations of phillyrin (0, 0.1, and 0.2 mM) for 24 h were measured using the Annexin V‐FITC and PI apoptosis kits from Elabscience in Wuhan, China. The cells that were gathered were suspended again in 500 μL of 1× binding buffer at a concentration of 1 × 10^6^ cells/mL. Then, the resuspended cells were dyed with Annexin V‐FITC and PI reagents in the dark at 4°C for 15–20 min. Phillyrin treatment effects on CRC cell apoptosis rate were evaluated using flow cytometry. Experiments were independently performed at least three times.

### 2.12 Cell Migration Assay

HT29 and HCT116 cells were placed in a six‐well plate and incubated in a cell culture chamber at 37°C until they reached 90% confluence. Subsequently, small pipette tips were employed to make scratches, and PBS was applied to wash off the exfoliated cells. Then the CRC cell lines were incubated with phillyrin (0, 0.1 mM, and 0.2 mM) and allowed to refill the gap. Images of the gap were captured every 24 h, and the healing percentage of the gap was calculated to measure the migration capacity of the cells. Experiments were independently performed at least three times.

### 2.13 Cell Invasion Assay

The cell invasion test was conducted by utilizing transwell chambers from Costar in New York, USA, where the upper chamber's basement membrane was covered with Matrigel. Cells were grown in the top chamber with a density of 5 × 10^4^ cells per chamber in 200 μL of DMEM medium without serum, whereas the bottom chamber had 600 μL of medium with 20% FBS added. Following a 24‐h incubation period with phillyrin at concentrations of 0, 0.1 mM, and 0.2 mM, any cells that did not migrate through the upper chamber were gently removed using cotton swabs. Following treatment with 4% formaldehyde and staining using 0.1% crystal violet, the quantity of infiltrating cells was tallied in five arbitrarily chosen areas through a microscope. Experiments were independently performed at least three times.

### 2.14 Western Blotting Analysis

Briefly, HT29 and HCT116 cells from each group were lysed to extract total protein using RIPA (Beyotime, Shanghai, China). The total protein was quantified and mixed with 5× loading buffer before boiling. A 20 μL sample containing 50 ng of total protein in loading buffer was separated by 10% SDS‐PAGE. After electrophoresis, the proteins were transferred to a PVDF membrane. After blocking with 5% skim milk, the membrane was incubated with primary antibodies overnight at 4°C. On the following day, the membrane was washed with TBST and then incubated with an HRP‐conjugated secondary antibody. To visualize the protein bands, chemiluminescence (ECL) was employed and exposed to radiographic film. The representative blot bands were repeated at least three times.

### 2.15 Statistical Analyses

The data are represented as the mean ± SD from at least three independent biological replicates. GraphPad Prism 9.0 software was utilized for statistical analyses, with normality test and one‐way ANOVA followed by Tukey's post hoc test used to determine significant differences between groups. A *p*‐value < 0.05 was considered statistically significant.

## 3 Results

### 3.1 The Collection of Drug Action Targets and CRC Relevance Targets

**FIGURE 2:**
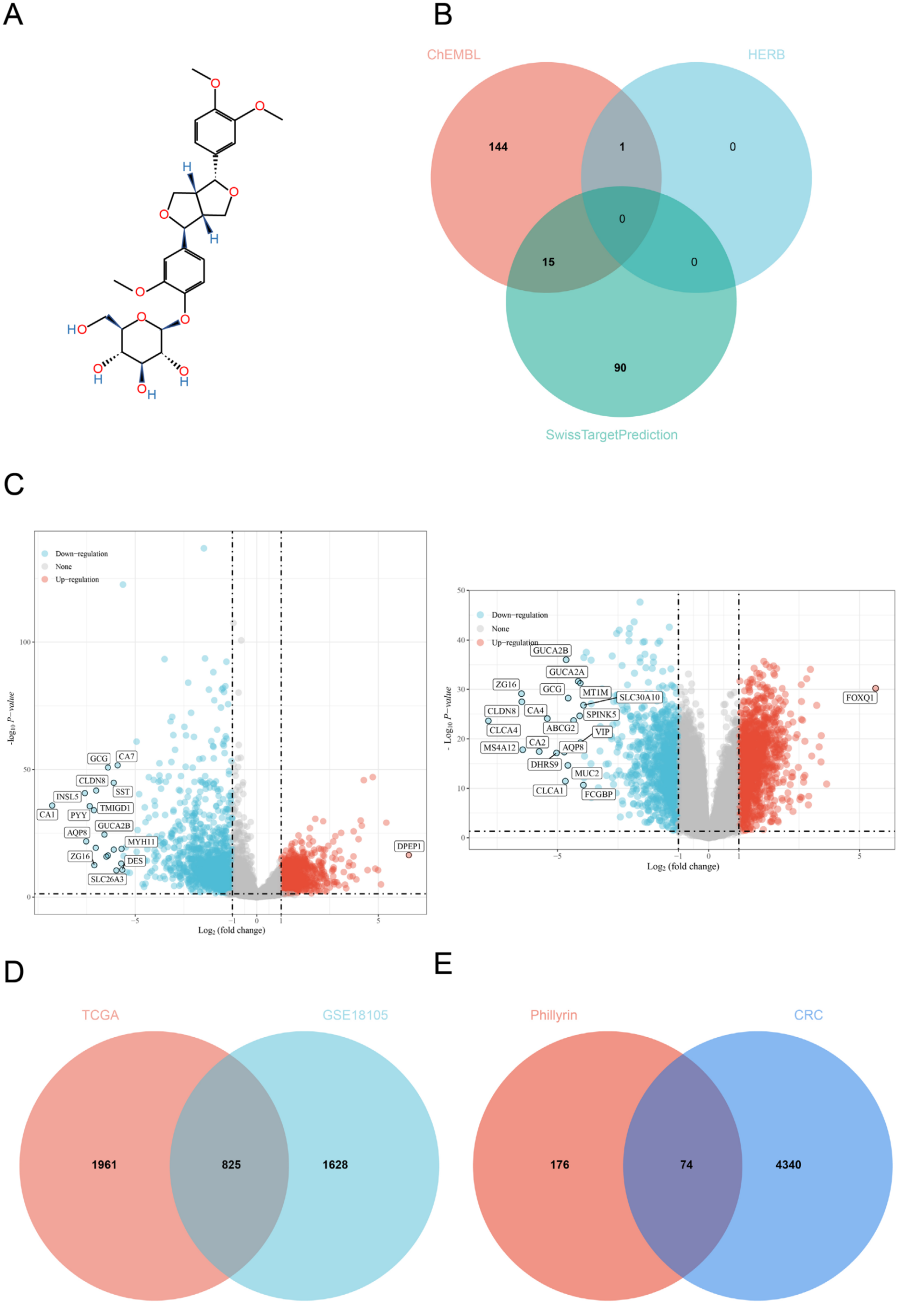
The collection of drug action targets and CRC relevance targets. (A) The chemical structure of phillyrin; (B) Venn diagram showing the related targets of phillyrin; (C) Volcano plots for differential gene expression of CRC from the TCGA and GEO databases; (D) Venn diagram of overlapping differential genes of CRC from the TCGA and GEO databases; (E) Venn diagram of phillyrin and CRC‐related targets.

The structural formula of phillyrin is shown in Figure 2A. To understand the potential therapeutic targets of phillyrin against CRC, we screened 250 potential target genes of phillyrin using the ChEMBL, HERB, and SwissTargetPrediction databases (Figure 2B). Similarly, CRC samples from the TCGA and GEO databases were used to identify differential genes (Figure 2C) and acquire a total of 4414 potential disease targets (Figure 2D). Subsequently, as illustrated in Figure 2E, a Venn diagram was drawn to display the intersection between phillyrin targets and CRC targets, yielding 74 drug‐disease overlapping genes for further investigation.

### 3.2 Creation of Compound‐Target‐Disease (CTD) Network Along With Protein–Protein Interaction (PPI) Network

**FIGURE 3:**
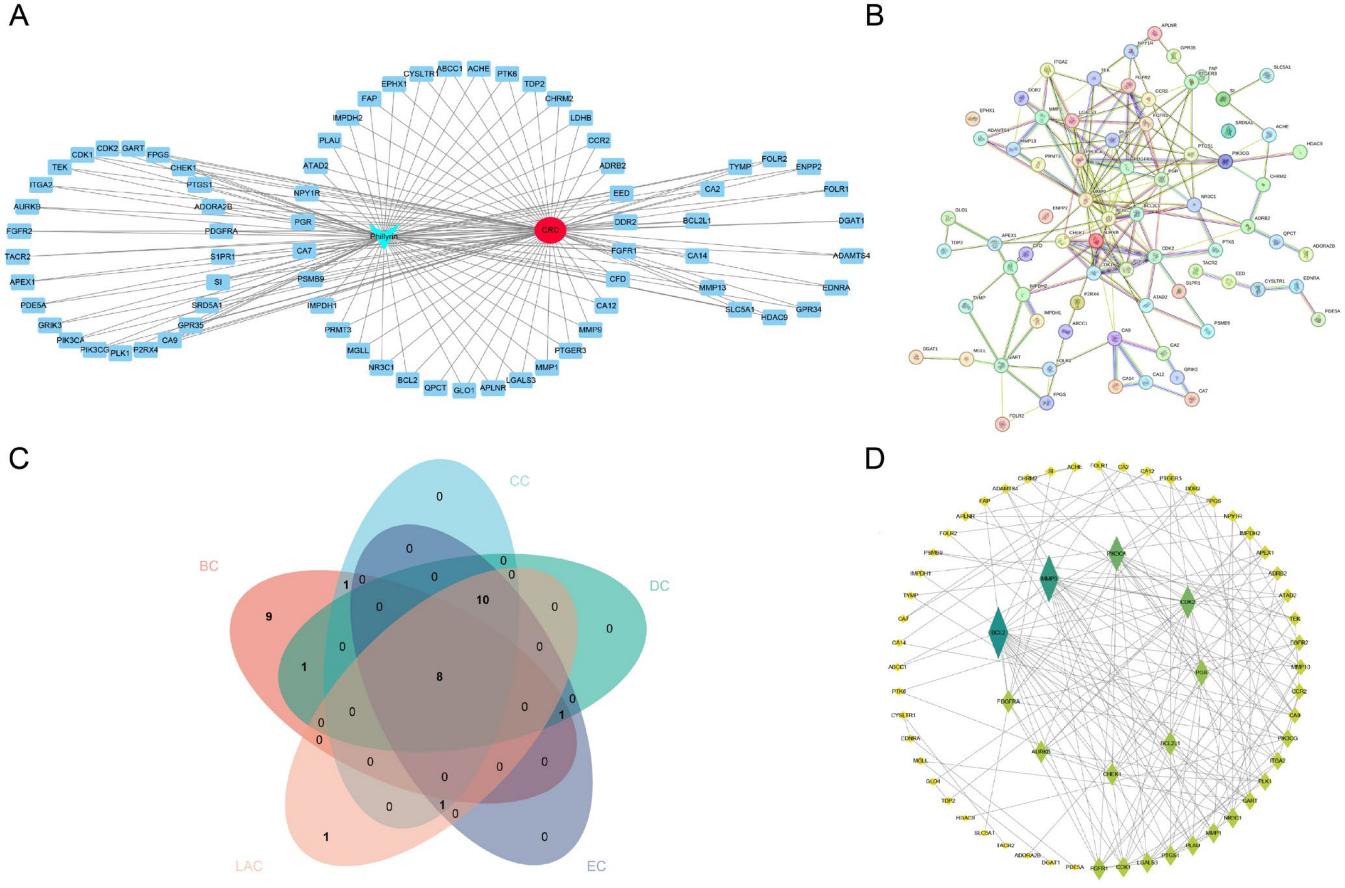
Establishment of CTD network and PPI network. (A) CTD network diagram; (B) PPI network diagram; (C) the Venn diagram illustrates the overlapping nodes with the highest scores for BC, CC, DC, EC, and LAC; (D) the PPI core target network. The node size is proportional to the degree value in the network.

Using Cytoscape software, we created a CTD network linking compounds, targets, and diseases to demonstrate phillyrin's pharmacological effectiveness in treating CRC (Figure 3A). This network comprised nodes representing the aforementioned 74 overlapping genes (in blue), phillyrin (in cyan), and CRC (in red). This finding suggested that phillyrin exerts tumor‐suppressive activity on colorectal cancer cells through multiple targets. Furthermore, a network of protein–protein interactions (PPI) was established using data from the STRING database to explore the potential ways in which phillyrin fights against CRC (Figure 3B). Subsequently, using Cytoscape software, we analyzed the topological properties of the PPI network and scored key network parameters such as BC, CC, DC, EC, and LAC for each node. The top 10 nodes were selected based on their scores. As shown in Figure 3C, a Venn diagram was generated for the top 20 nodes in each of the five categories of topological features, and we identified eight hub genes, namely Bcl2, MMP9, CDK2, PIK3CA, PDGFRA, AURKB, NR3C1, and PGR. Furthermore, we established a PPI core target network (Figure 3D).

### 3.3 GO Enrichment and KEGG Pathway Analysis

**FIGURE 4:**
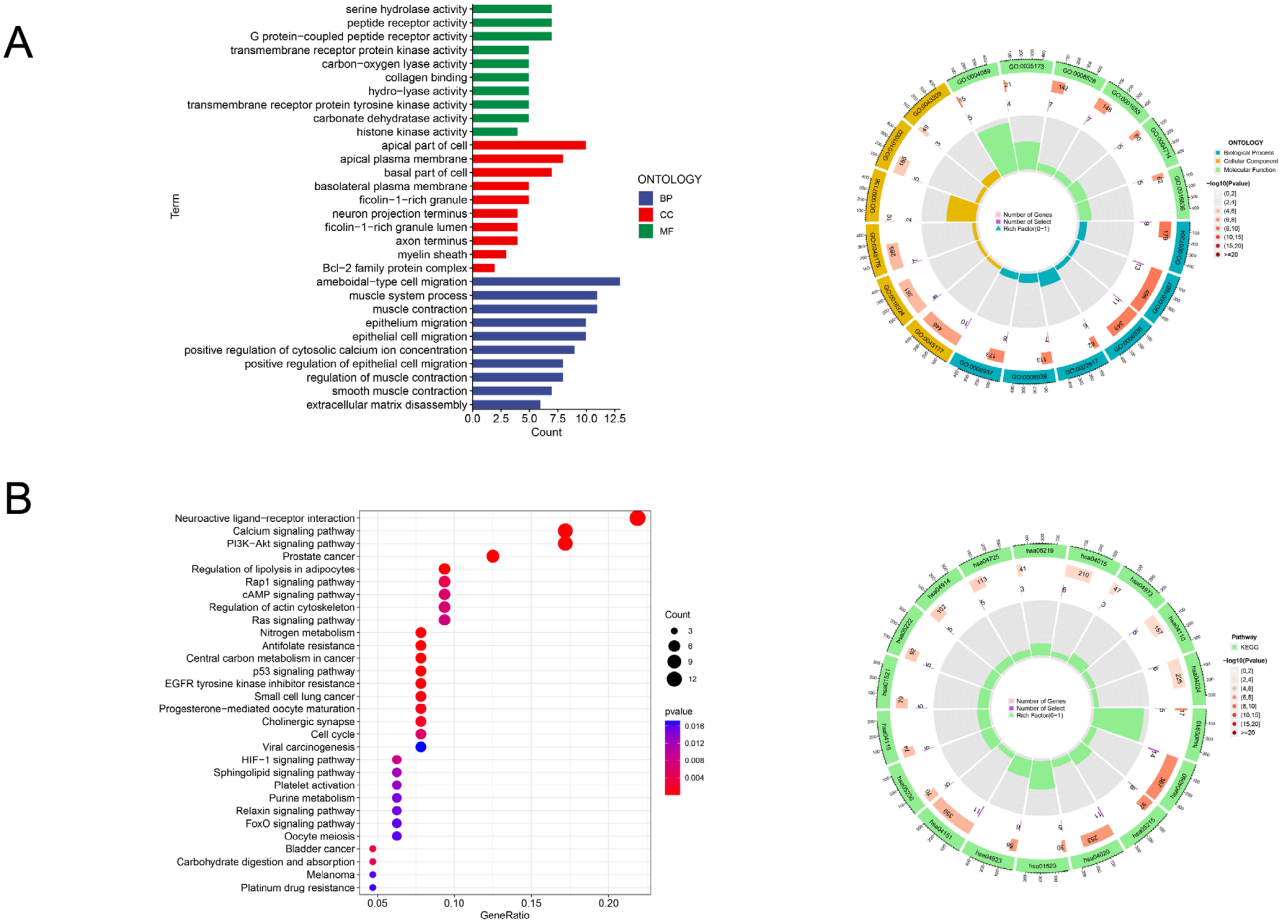
GO enrichment and KEGG pathway analysis. (A) GO enrichment analysis. The bar chart displays the top ten enrichment analyses; blue, red, and green represent the enrichment analyses of BP, CC, and MF, respectively. The pie charts on the right display the enrichment scores of the top six GO terms in each category; (B) KEGG enrichment analysis. Bubble diagram illustrates the top 30 enriched pathways. The pie charts on the right display the enrichment scores of the top 16 pathways.

We performed GO and KEGG enrichment analyses on shared genes to evaluate how phillyrin affects biological processes and signaling pathways involved in the development and advancement of CRC. The analysis of GO annotations included three classifications: biological processes (BP), cellular components (CC), and molecular functions (MF). Bar graphs illustrated the notably enriched terms for biological processes, cellular components, and molecular functions, with pie charts on the right showing the enrichment scores of the top six Gene Ontology terms in each category (Figure 4A). The results of the GO enrichment analysis suggested that MF is mainly related to peptide receptor function and transmembrane receptor protein kinase function. BP encompassed processes such as ameboidal‐type cell migration and extracellular matrix disassembly, both of which are associated with tumor cell migration and invasion. Our findings indicated that phillyrin may regulate the biological activities of tumor cells through various pathways involving protein kinases and related transmembrane receptors, potentially exerting pharmacological effects on tumor growth and metastasis.

Likewise, KEGG enrichment analysis was conducted on overlapping genes, and bar charts were generated to illustrate the significantly enriched 30 pathways, filtered based on *p*‐value and gene ratio. The enrichment scores of the top 16 pathways were shown in the pie charts to the right (Figure 4B). The KEGG enrichment analysis showed a notable enrichment of shared genes in various pathways associated with tumor cell advancement, particularly the PI3K/AKT pathway, Ras signaling pathway, p53 signaling pathway, and prostate cancer pathway. In summary, the enrichment‐based results highlight the close association of core targets with multiple signaling pathways, with the PI3K/AKT pathway showing significant enrichment.

### 3.4 Molecular Docking and Molecular Dynamics Simulation

**FIGURE 5:**
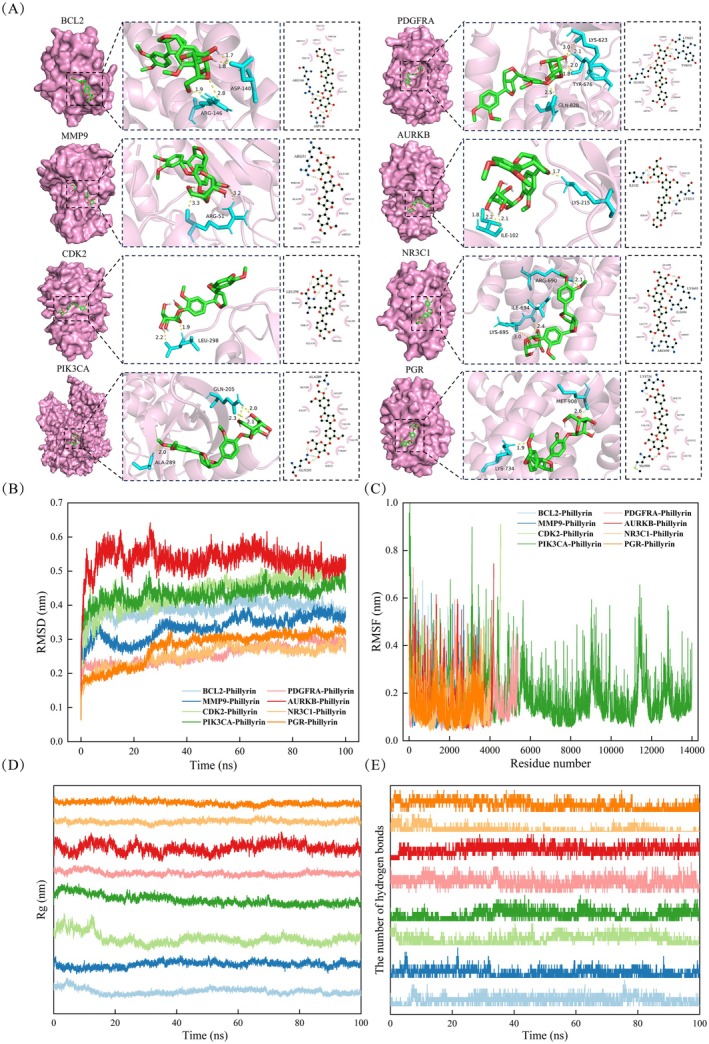
Molecular docking and molecular dynamics simulation. (A) Molecular docking between phillyrin and the proteins BCL2, MMP9, CDK2, PIK3CA, PDGFRA, AURKB, NR3C1, and PGR. And analysis of molecular dynamics simulation for (B) RMSD; (C) RMSF; (D) RG; (E) the number of hydrogen bonds.

To further validate the interaction between phillyrin and the hub targets, we conducted molecular docking of the ligand (phillyrin) with the receptors. The binding free energy of the complexes was used to quantify the affinity between phillyrin and the eight hub targets obtained. The optimal conformations from molecular docking were visualized using PyMOL, as shown in Figure 5A. Analysis of 50 blind docking results revealed the binding free energies of BCL2, MMP9, CDK2, PIK3CA, PDGFRA, AURKB, NR3C1, and PGR with phillyrin to be −5.95, −6.26, −4.93, −4.51, −6.09, −5.25, −4.6, and −5.81 kcal/mol, respectively (Tables 1 and 2). The docking binding energies for these molecules were all less than −4.5 kcal/mol, indicating good binding capability between phillyrin and these hub targets.

**TABLE 1:** Optimal conformational interaction energy rules.

	ΔG/(Kcal/mol)	Eintermol/(Kcal/mol)	Evhd/(Kcal/mol)	Eelec/(Kcal/mol)
BCL2‐Phillyrin	−5.95	−9.53	−8.71	−0.82
MMP9‐Phillyrin	−6.26	−9.84	−9.76	−0.08
CDK2‐Phillyrin	−4.93	−8.51	−8.28	−0.23
PIK3CA‐Phillyrin	−4.51	−8.09	−8.05	−0.05
PDGFRA‐Phillyrin	−6.09	−9.67	9.56	−0.12
AURKB‐Phillyrin	−5.25	−8.83	−8.48	−0.35
NR3C1‐Phillyrin	−4.60	−8.18	−7.91	−0.27
PGR‐Phillyrin	−5.81	−9.39	−9.07	−0.31

**TABLE 2:** Binding energy results of the first 10 molecular dockings.

	1	2	3	4	5	6	7	8	9	10
BCL2‐Phillyrin	−5.95	−5.93	−5.62	−5.54	−5.3	−5.26	−5.24	−5.14	−5.10	−4.96
MMP9‐Phillyrin	−6.26	−5.99	−5.59	−5.38	−5.33	−5.19	−5.18	−5.02	−5.01	−4.73
CDK2‐Phillyrin	−4.93	−4.52	−4.48	−4.22	−3.98	−3.86	−3.74	−3.66	−3.65	−3.63
PIK3CA‐Phillyrin	−4.51	−4.27	−4.08	−4.07	−3.94	−3.9	−3.6	−3.48	−3.36	−3.36
PDGFRA‐Phillyrin	−6.09	−6.05	−4.92	−4.16	−4.13	−4.12	−4.06	−4.04	−4.03	−4.02
AURKB‐Phillyrin	−5.25	−5.23	−4.89	−4.87	−4.71	−4.65	−4.59	−4.55	−4.42	−4.33
NR3C1‐Phillyrin	−5.81	−5.75	−5.51	−5.14	−5.13	−5.03	−4.96	−4.89	−4.48	−4.47
PGR‐Phillyrin	−4.60	−4.24	−4.17	−4.16	−4.11	−4.01	−4.00	−3.88	−3.86	−3.84

In addition, to validate the results of molecular docking, 100 ns molecular dynamics simulations were performed. The results showed that all eight complexes exhibited relatively stable RMSD values and minimal fluctuations (< 0.1 nm, 1.0 Å) within 100 ns, indicating that the binding of the complexes was relatively stable (Figure 5B). This structural stability suggests that phillyrin maintained a consistent conformation at the binding site, confirming the reliability of the aforementioned interaction modes. RMSF values quantified the structural stability and atomic mobility of the proteins, and the results indicated that the binding of phillyrin stabilized the internal fluctuations of the proteins, thereby contributing to potential pharmacological properties, while the overall structures of the complexes remained relatively stable (Figure 5C). The radius of gyration (Rg), an important indicator for evaluating the compactness and stability of protein–ligand complexes, remained largely stable across all eight complexes, suggesting stable conformations and compact folding (Figure 5D). Hydrogen bonding is a key indicator of the binding strength between proteins and ligands; the results showed that all eight complexes exhibited stable hydrogen bond patterns (Figure 5E). The orderly hydrogen bond patterns are critical for maintaining the stability of the complexes, indicating that phillyrin exerts a significant regulatory effect on key CRC targets and may serve as an effective therapeutic agent for CRC. Compared with the other seven complexes, the PIK3CA–phillyrin complex exhibited more stable RMSD, RMSF, Rg, and hydrogen bond numbers, indicating that phillyrin effectively targets PIK3CA and exerts a significant regulatory effect on the PI3K/AKT signaling pathway.

### 3.5 Phillyrin Inhibits Colorectal Cancer Cell Viability and Induced Apoptosis

**FIGURE 6:**
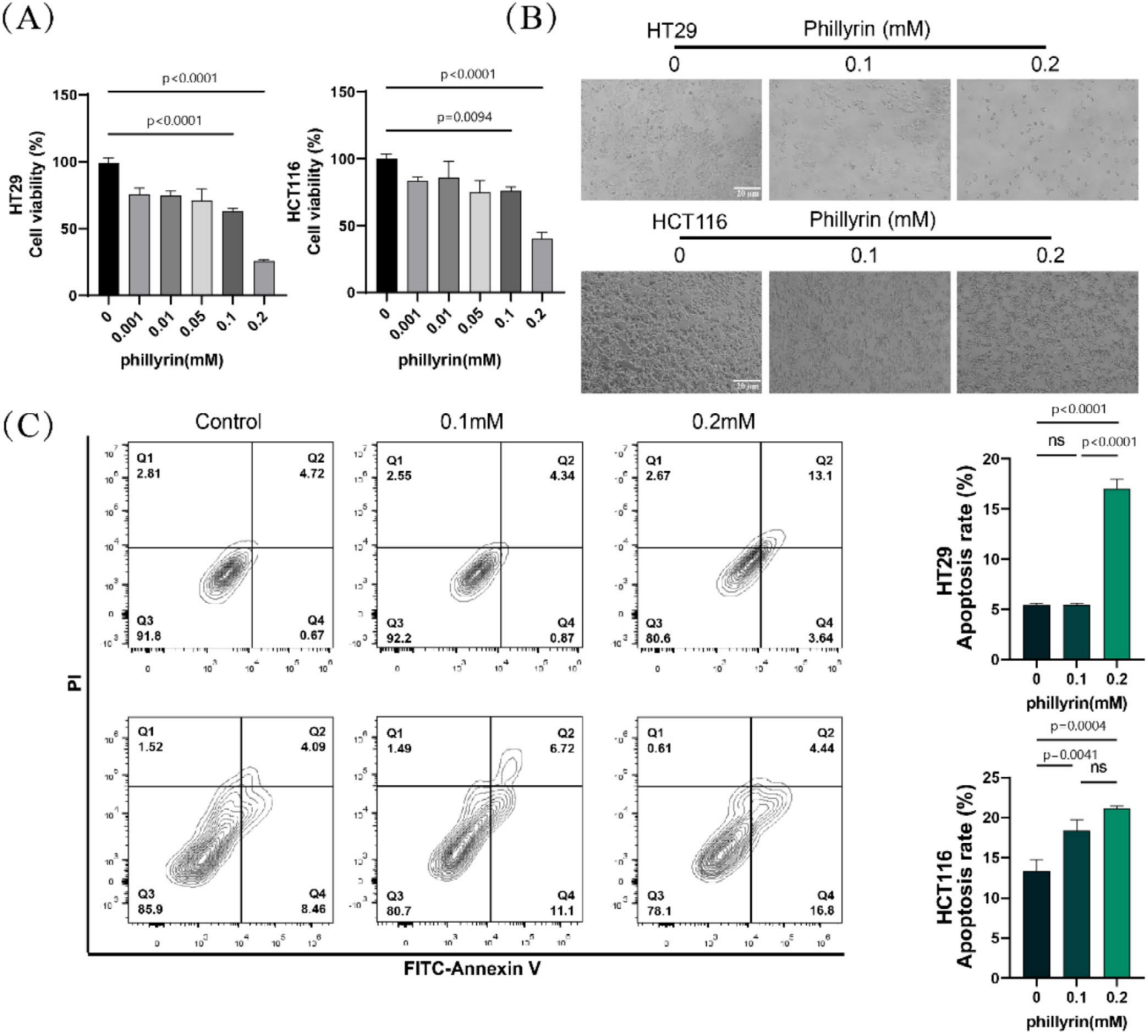
Phillyrin inhibits colorectal cancer cell viability and induces apoptosis. (A) The cell viability of HT29 and HCT116 was detected by CCK8 assays. Values are expressed as means ± SD; *n* = 3. *p < 0.05, **p < 0.01, ***p < 0.001 vs. control group. (B) The live cells were photographed after being treated with phillyrin for 24 h. (C) Flow cytometry of apoptosis was applied to detect apoptosis ratio of colorectal cancer cells after being incubated with phillyrin. Apoptosis ratio = (Q2 + Q3)/(Q1 + Q2 + Q3 + Q4). Values are expressed as means ± SD; *n* = 3. *p < 0.05, **p < 0.01, ***p < 0.001 vs. control group (*n* = 3).

We conducted CCK‐8 assays and apoptosis flow cytometry analysis to evaluate how phillyrin affects the survival of colorectal cancer cells. The results indicated that phillyrin inhibited the growth of HT29 and HCT116 cells in a concentration‐dependent manner, significantly reducing cell viability at concentrations of 0.1 mM and 0.2 mM (Figure 6A). Following treatment with phillyrin, a reduction in the quantity of HT29 and HCT116 cells was noted, along with an increase in the distance between cells and cell contraction (Figure 6B).

To determine whether the phillyrin‐mediated inhibition of colorectal cancer cell proliferation was associated with apoptosis, flow cytometry analysis demonstrated that phillyrin significantly promoted apoptosis, particularly at 0.2 μM, where apoptosis rates in HT29 and HCT116 cells markedly increased (Figure 6C). Consistently, the upregulation of Bax and downregulation of Bcl2 further confirmed this effect (Figure S1A,B). These results indicated that phillyrin is promising in hindering the growth of colorectal cancer cells and promoting cell death.

### 3.6 Phillyrin Attenuates the Cells Proliferation and Metastasis of CRC

**FIGURE 7:**
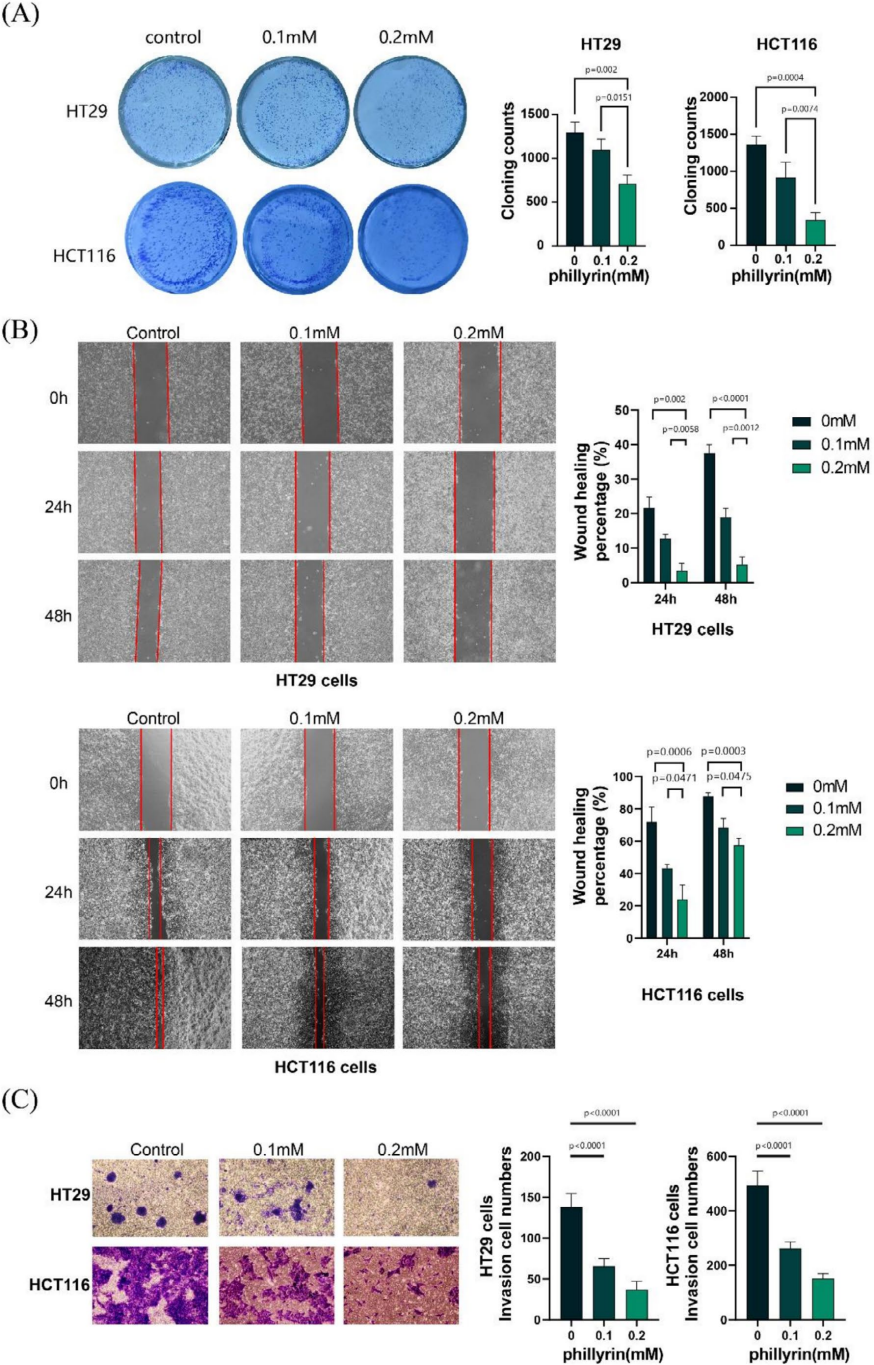
Phillyrin attenuates the cells' proliferation and metastasis of CRC. (A) Colony formation assays displayed the proliferation of colorectal cancer cells was suppressed by phillyrin in a dose‐dependent manner. (B) Wound healing assay demonstrated the effect of phillyrin on the migration ability of HT29 and HCT116. *p < 0.05. (C) Transwell assay suggested that the colorectal cancer cell invasion was significantly repressed by phillyrin. Values are expressed as means ± SD; *n* = 3. *p < 0.05, **p < 0.01, ***p < 0.001 vs. control group (*n* = 3).

The primary antitumor effect of phillyrin is mediated through the inhibition of colorectal cancer cell proliferation, migration, and invasion. As presented in Figure 7A, the colony formation assay demonstrated that phillyrin treatment significantly reduced the number of colonies formed by HT29 and HCT116 cells. Hence, phillyrin exhibits significant proliferation inhibition against CRC.

The epithelial‐mesenchymal transition (EMT) plays a critical role in the progression of CRC, facilitating its metastasis and contributing to cancer development and poor prognosis, partly through hematogenous dissemination. To assess phillyrin's potential impact on colorectal cancer cell migration ability, we conducted scratch assays. After treatment with phillyrin (0, 0.1, and 0.2 mM) for 24 h, HT29 and HCT116 cells exhibited significantly reduced migration rates in a concentration‐dependent manner (Figure 7B). Besides, we performed transwell assays to evaluate the invasion ability of colorectal cancer cells. As shown in Figure 7C, HT29 and HCT116 cells in the control groups exhibited robust invasive capabilities, whereas phillyrin treatment markedly suppressed their invasion. Phillyrin has been shown to effectively block the growth of colorectal cancer cells in a dose‐dependent manner, as well as suppress the process of EMT.

### 3.7 Phillyrin Inhibits the PI3K/AKT/mTOR Signaling Pathway of CRC

**FIGURE 8:**
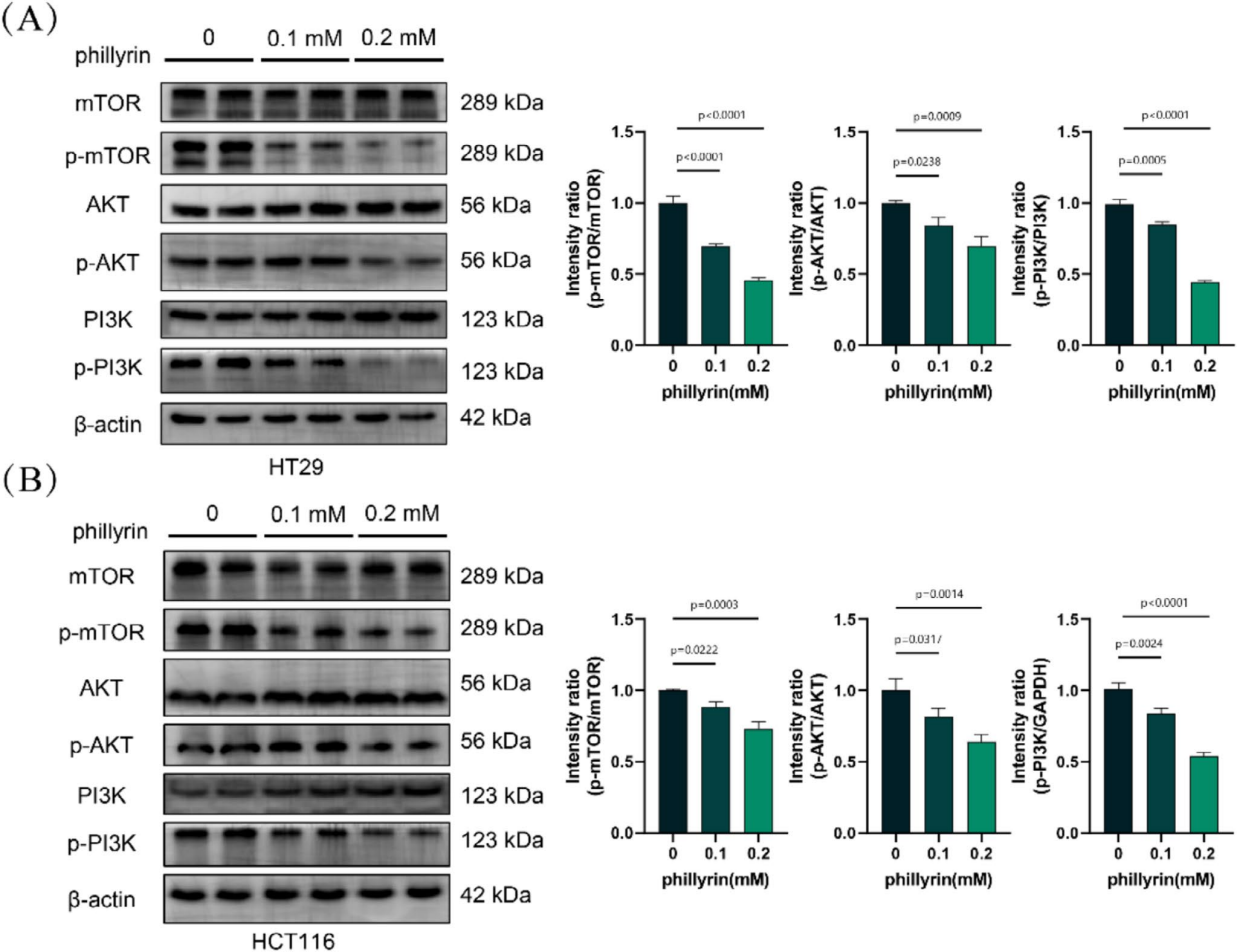
Phillyrin inhibits the PI3K/AKT/mTOR signaling pathway. (A, B) The western blot analysis revealed a downregulation in the expression of p‐PI3K, p‐AKT, and p‐mTOR in HT29 and HCT116 cells following treatment with phillyrin. Values are expressed as means ± SD; *n* = 3. *p < 0.05, **p < 0.01, ***p < 0.001 vs. control group (*n* = 3).

As mentioned previously, the PI3K/AKT signaling pathway was identified as a significantly enriched target among the potential mediators of phillyrin's effects in CRC. It is a critical signaling pathway involved in tumor development, regulating cell survival, migration, and metabolism, and playing a role in angiogenesis and the recruitment of inflammatory factors. We conducted Western blot analysis to examine the effects of phillyrin on the PI3K/AKT signaling pathway in colorectal cancer cells by assessing the levels of PI3K, p‐PI3K, AKT, p‐AKT, mTOR, and p‐mTOR, along with their downstream targets, in HT29 and HCT116 cells. As shown in Figure 8, the expression of p‐PI3K, p‐AKT, and p‐mTOR in cells was downregulated by phillyrin in a dose‐dependent manner, while showing no significant effect on the expression of PI3K, AKT, and mTOR.

To further verify that phillyrin exerts its antitumor effects through the PI3K/AKT signaling pathway, we performed PI3K overexpression experiments. The results showed that PI3K overexpression attenuated the inhibitory effect of phillyrin on tumor cell viability (Figure S2A), reversed its suppression of cell proliferation (Figure S2B), and restored the migratory and invasive capacities of CRC (Figure S2C,D). Thus, we proposed that phillyrin hinders PI3K/AKT/mTOR signaling pathway activation, decreases mTOR downstream effector activation, and ultimately inhibits colorectal cancer proliferation and EMT progression.

## 4 Discussion

CRC is the third most prevalent cancer in the digestive tract, known for its genetic diversity, tendency to spread, and fast advancement. Moreover, the overall 5‐year survival rate for advanced‐stage CRC patients is less than 10% (Siegel et al. 2017; Yaeger et al. 2018). The occurrence of metastasis in CRC patients often indicates an advanced disease stage, serving as a major cause of patient mortality (Stella Tsai et al. 2010). Current treatment modalities for CRC mainly consist of surgical resection and chemotherapy adjuncts with 5‐fluorouracil (Brenner et al. 2014). Despite some progress in CRC treatment in recent years (Biller and Schrag 2021), particularly yielding favorable outcomes in early‐stage patients, the emergence of drug resistance hampers the efficacy of existing treatment regimens, resulting in insufficient improvement in patient survival rates. Therefore, novel therapeutic agents are urgently needed to enhance the treatment of CRC.

Extensive research has demonstrated the diverse pharmacological benefits of phillyrin, including its anti‐inflammatory, antiviral, and antiaging properties (Tang et al. 2022; Zhou et al. 2022). For instance, in traumatic brain injury, phillyrin inhibits the phosphorylation of NF‐κB, activates the PPARγ signaling pathway, and suppresses microglia‐induced inflammatory damage (Jiang et al. 2020). The continuous existence of inflammatory cells and their corresponding chronic inflammatory responses plays a vital role in the tumor microenvironment, serving as key catalysts in the development and advancement of tumors (Dmitrieva‐Posocco et al. 2019). Therefore, phillyrin also holds significant potential in cancer therapy. Furthermore, research by (Xu et al. 2024) has confirmed that phillyrin can induce cell autophagy, thereby treating nasopharyngeal carcinoma (Wang et al. 2019), suggesting its potential antitumor effects. Research indicates that in colorectal cancer, disruption of the mucosal barrier results in the translocation of pro‐inflammatory mediators, which triggers the activation of the interleukin (IL)‐23–IL‐17A signaling pathway. The increased cytokines induced often indicate a poor prognosis (Tosolini et al. 2011). However, there is currently limited research on the effects of phillyrin on CRC. Hence, this study aims to investigate the possible anticancer effects of phillyrin on colorectal cancer. The network pharmacology and molecular docking techniques were used to explore the underlying mechanisms of phillyrin, followed by validation of the docking results using molecular dynamics simulations. Additionally, based on the analysis of key targets, GO terms, and KEGG pathways' complex interactions, we determined the important functions of phillyrin and its anti‐CRC targets. Additionally, we confirmed phillyrin's anti‐CRC activity in a laboratory setting and its impact on important signaling pathways.

CRC is one of the top contributors to cancer‐related fatalities worldwide, known for its high rates of recurrence and metastasis. Tumor metastasis, considered a late‐stage hallmark, is the primary cause of mortality in CRC patients (Liu et al. 2024). The spread of cancer cells from the original tumor to different areas, often through direct invasion, the bloodstream, or lymphatic pathways, leads to the presence of cancer cells in nearby tissues or distant organs, a process known as tumor metastasis (Bielenberg et al. 2004). The process of metastasis involves a complex interplay of cellular and molecular events. EMT is an early event in tumor metastasis and a key mechanism in CRC metastasis and invasion (Ma et al. 2021). During CRC progression, EMT causes tumor cells to lose their epithelial traits, such as cell polarity, intercellular connections, and basement membrane integrity, ultimately promoting their ability to migrate and invade surrounding tissues (He et al. 2022). In our study, we conducted GO functional analysis of the key targets of phillyrin and found that phillyrin affects the processes of epithelial cell migration and extracellular matrix degradation. Collagen, as a major component of the extracellular matrix, provides support for tumor cell migration during EMT, promoting their movement and invasion (Jiang et al. 2022). GO functional analysis also suggests that phillyrin may affect collagen binding in CRC cells, thereby inhibiting CRC metastasis. Additionally, through in vitro experiments, we validated the significant effects of phillyrin on inhibiting the metastatic capabilities of CRC cells.

In addition to aberrant migration and invasion activities, resistance to apoptosis, abnormal proliferation, and dysregulated cell signaling are also important hallmarks of tumor progression (Deuker et al. 2015; Li et al. 2022). Through studying the potential target genes of phillyrin in treating CRC, we constructed a PPI network and, based on topological evaluation, identified eight key target genes: Bcl2, PDGFRA, MMP9, AURKB, CDK2, NR3C1, PIK3CA, and PGR. The importance of Bcl‐2 in controlling cell apoptosis is widely acknowledged. Overexpression of Bcl‐2 in tumors may lead to resistance of cancer cells to apoptosis, thereby promoting tumor growth and survival (Yan et al. 2022). The cyclin‐dependent kinase CDK2 is essential for regulating the transition from the G1 to S phases in the cell cycle. Aberrant activation of CDK2 is often associated with the uncontrolled proliferation of tumor cells (Zhang et al. 2022). Additionally, PDGFRA and AURKB, as important protein kinases in cell signaling pathways, play significant roles in regulating growth factor signaling and the process of cell mitosis (Cho et al. 2012; Nagano et al. 2021). Therefore, modulating these hub nodes may help suppress tumor development. To investigate the regulation of these target genes by phillyrin, we conducted molecular docking and validated the obtained interaction patterns within the complexes through molecular dynamics simulations.

PIK3CA is responsible for producing the p110 catalytic subunit of PI3K, which plays a vital role in the PI3K/AKT signaling pathway that controls important biological functions such as cell metabolism, growth, and viability (Giedt et al. 2018; Zheng et al. 2023). Studies have demonstrated that mutations and aberrant activation of PIK3CA are associated with the progression of various cancers (Fruman and Rommel 2014). Upon abnormal activation, PI3K promotes the conversion of PIP2 to PIP3, thereby resulting in AKT hyperactivation. Activated AKT phosphorylates and modifies various downstream target proteins, including cell cycle regulatory proteins and transcription factors, promoting cell proliferation and growth (Chen et al. 2018). AKT also inhibits apoptosis‐related proteins, such as members of the caspase families and Bcl‐2 families, through phosphorylation. This process blocks apoptosis signal transduction and imparts resistance to apoptosis in tumor cells (Jiang et al. 2019). Furthermore, the PI3K/AKT pathway controls the production of vascular endothelial growth factor, which enhances the proliferation and movement of endothelial cells, aiding in the delivery of necessary nutrients and oxygen for tumor development (Liu et al. 2019). mTOR, a key kinase downstream of the PI3K/AKT signaling axis, plays a crucial role in regulating diverse biological processes, including cell growth, metabolism, and proliferation. Aberrant activation of mTOR is often detected in some types of tumors (Wagle et al. 2014). Additionally, mTOR boosts the capacity of tumor cells to invade and metastasize through its control over the restructuring of the cellular cytoskeleton and the production of matrix metalloproteinases. Therefore, targeting mTOR inhibition is believed to inhibit tumor progression (Wang et al. 2014). Based on our research findings, phillyrin can interact with PIK3CA by binding GLN205, TYR207, TYR250, and ALA289. These amino acid residues are situated within the phosphatidylinositol 3‐kinase Ras‐binding domain of PIK3CA. Therefore, we suggest that phillyrin interacts with PI3K, occupying its functional domain and ultimately inhibiting the activation of downstream signals. Our research further validated this theory by showing a significant decrease in the levels of p‐AKT and p‐mTOR in HT29 and HCT116 cells after being exposed to phillyrin.

In addition to the PI3K/AKT signaling pathway, KEGG enrichment analysis also revealed significant enrichment in pathways such as p53 and Ras, suggesting that phillyrin may exert broader regulatory effects through multiple signaling cascades. The PI3K/AKT and p53 pathways are known to interact extensively, as activation of PI3K/AKT can suppress p53‐mediated apoptosis by regulating downstream effectors, such as MDM2, thereby promoting cell survival. Conversely, p53 can negatively regulate PI3K/AKT signaling by transcriptionally modulating PTEN expression, indicating a reciprocal regulatory loop. Similarly, the Ras signaling pathway represents an important upstream regulator of PI3K/AKT, and aberrant Ras activation can strongly drive PI3K/AKT‐mediated oncogenic signaling. These findings suggest that the antitumor effects of phillyrin may not be limited to a single pathway but rather involve coordinated modulation of a signaling network in which PI3K/AKT acts as a central hub. In the follow‐up study, we will explore these interactions to provide a more comprehensive understanding of phillyrin's molecular mechanisms in CRC.

Despite the promising findings of this study, several limitations should be acknowledged. First, only two CRC cell lines were employed, which may not fully capture the heterogeneity of colorectal cancer, and in vivo experiments were not conducted to validate the therapeutic effects of phillyrin in a more physiological context. Second, although the affinity of 5‐fluorouracil (5‐FU) for its target appears weaker than that of phillyrin, this study did not conduct a direct comparison of their therapeutic efficacy. Therefore, it remains challenging to evaluate the relative efficacy and potential clinical advantages of phillyrin. Moreover, as a natural product, phillyrin suffers from pharmacokinetic limitations, including low oral bioavailability and rapid metabolism, which may restrict its therapeutic application. In future studies, we will conduct experiments in vivo to confirm its efficiency, as well as explore structural modification or advanced delivery systems to improve its bioavailability and tissue distribution, which will be essential for advancing phillyrin from experimental research toward clinical application. Furthermore, phillyrin could be optimized as a lead compound and evaluated in combination with standard CRC therapies to enhance antitumor efficacy. Such translational strategies may facilitate its progression toward preclinical and clinical development, highlighting its potential as a novel therapeutic option for colorectal cancer.

## 5 Conclusion

In this study, we creatively integrated network pharmacology, molecular docking, and molecular dynamics simulations to systematically elucidate the potential mechanisms of phillyrin in CRC. Bioinformatics analyses indicated that phillyrin may exert therapeutic effects by modulating key targets, including Bcl2, CDK2, PDGFRA, AURKB, and PIK3CA. Experimental validation further demonstrated that phillyrin significantly suppressed tumor cell viability and metastasis while promoting apoptosis. Moreover, phillyrin was found to inhibit the PI3K/AKT/mTOR signaling pathway, highlighting its antagonistic role in CRC progression. Collectively, these findings reveal the pharmacological potential of phillyrin against CRC, provide mechanistic insights into its anticancer activity, and establish a theoretical basis for its future clinical development.

## Author Contributions

Z.‐J.H. performed formal analysis and writing the original draft. G.‐J.L. contributed to data curation and reviewing the manuscript. W.‐S.D., F.Z., and P.‐H.Y. performed the analyses. S.‐L.Y., D.‐N.H., and B.‐Y.L. carried out visualization. Y.‐Y. Gao, D.‐Y.S., and Y.‐Y. Guo contributed to the methodology of the study. H.‐Y.Z. and Y.‐H.B. designed the study and revised the manuscript. All authors agree to be accountable for all aspects of the work.

## Conflicts of Interest

The authors declare no conflicts of interest.

## Supporting information


**Figures S1–S2:** fsn371069‐sup‐0001‐FigreS1‐S2.docx.

## Data Availability

All data supporting the findings of this study are available in the article and its Figures S1 and S2. For additional information, inquiries can be addressed to the corresponding author.
