# Supplementary material for: Network Pharmacology and Molecular Docking Elucidate the Mechanism of Phillyrin in Colorectal Cancer
Source: Food Sci Nutr. 2025 Oct 23;13(10):e71069. doi: 10.1002/fsn3.71069 (PMC12550133; doi:10.1002/fsn3.71069)
Supplement: Supplementary file 1 — Figures S1–S2: fsn371069‐sup‐0001‐FigreS1‐S2.docx. [file FSN3-13-e71069-s001.docx]

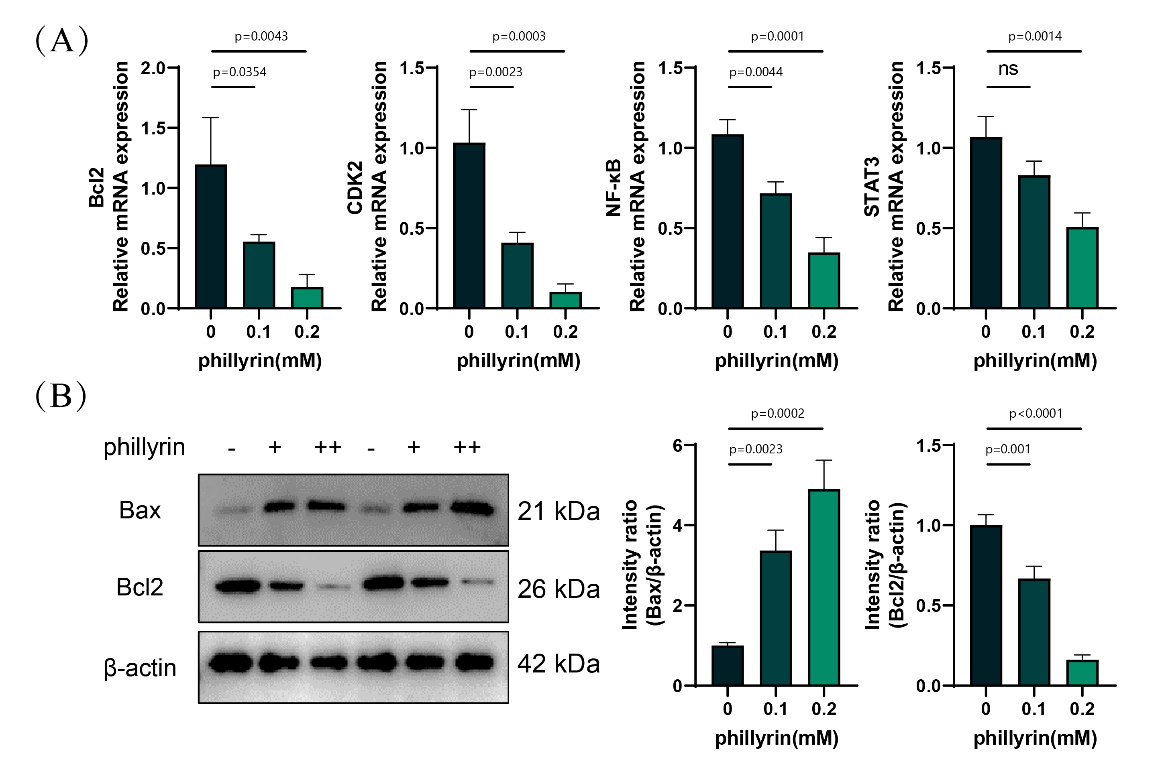


**FIGURE S1**

(A) Real-time PCR analyses of Bcl2, CDK2, NF-κB and STAT3 mRNA expression. (B) The Western blot analysis revealed an upregulation in the expression of Bax and downregulation of Bcl2 in cells following treatment with phillyrin. Values are expressed as means ± SD; n = 3. **P* < 0.05, ***P* < 0.01, ****P* < 0.001 vs control group (n=3)


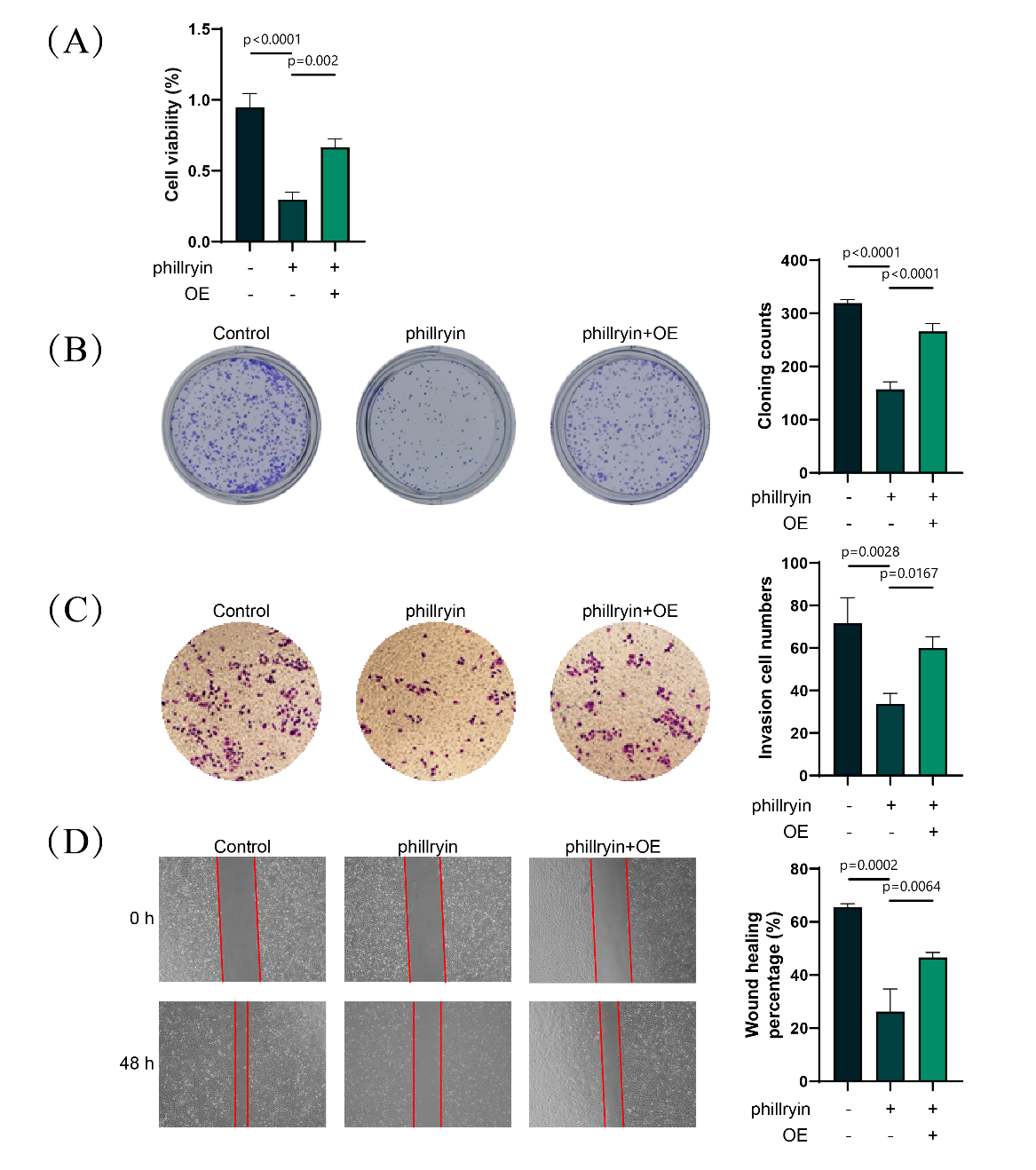


**FIGURE S2**

(A) Cell viability was assessed by CCK-8 assay. (B) Colony formation assay showed that PI3K overexpression reversed the phillyrin-induced suppression of proliferation. (C) Transwell assay indicated that PI3K overexpression reserved the inhibitory effect of phillyrin on invasion. (D) Wound healing assay demonstrated that PI3K overexpression counteracted the phillyrin-mediated inhibition of migration. **P* < 0.05. Values are expressed as means ± SD; n = 3. **P* < 0.05, ***P* < 0.01, ****P* < 0.001 vs control group (n=3).
